# The role of declining ataxia-telangiectasia-mutated (ATM) function in oocyte aging

**DOI:** 10.1038/s41420-024-02041-z

**Published:** 2024-06-25

**Authors:** Reiko Suzuki, Xiujuan Tan, Katarzyna J. Szymanska, Nada Kubikova, Columba Avila Perez, Dagan Wells, Kutluk H. Oktay

**Affiliations:** 1 Department of Obstetrics, Gynecology and Reproductive Sciences, Yale University School of Medicine, New Haven, USA; 2 Nuffield Department of Women’s and Reproductive Health, University of Oxford, Oxford, United Kingdom; 3 Juno Genetics, Oxford, United Kingdom

**Keywords:** Ageing, Cell growth, Embryogenesis

## Abstract

Despite the advances in the understanding of reproductive physiology, the mechanisms underlying ovarian aging are still not deciphered. Recent research found an association between impaired ATM-mediated DNA double-strand break (DSB) repair mechanisms and oocyte aging. However, direct evidence connecting ATM-mediated pathway function decline and impaired oocyte quality is lacking. The objective of this study was to determine the role of ATM-mediated DNA DSB repair in the maintenance of oocyte quality in a mouse oocyte knockdown model. Gene interference, in vitro culture, parthenogenesis coupled with genotoxicity assay approaches, as well as molecular cytogenetic analyses based upon next-generation sequencing, were used to test the hypothesis that intact ATM function is critical in the maintenance of oocyte quality. We found that ATM knockdown impaired oocyte quality, resulting in poor embryo development. ATM knockdown significantly lowered or blocked the progression of meiosis in vitro, as well as retarding and reducing embryo cleavage after parthenogenesis. After ATM knockdown, all embryos were of poor quality, and none reached the blastocyst stage. ATM knockdown was also associated with an increased aneuploidy rate compared to controls. Finally, ATM knockdown increased the sensitivity of the oocytes to a genotoxic active metabolite of cyclophosphamide, with increased formation of DNA DSBs, reduced survival, and earlier apoptotic death compared to controls. These findings suggest a key role for ATM in maintaining oocyte quality and resistance to genotoxic stress, and that the previously observed age-induced decline in oocyte ATM function may be a prime factor contributing to oocyte aging.

## Introduction

Ovarian aging, a culmination of both the decline in the quantity and quality of oocytes, affects every female. With aging, ovarian reserve and fertility decline, pregnancy failures, and oocyte and embryo aneuploidy gradually increase. In addition, the decline in follicle reserve in humans seems to accelerate after ages 36–37 [1]. The decreased fecundability of oocytes and increased pregnancy losses associated with chromosomal errors seen in later reproductive years, often referred to as diminished oocyte quality, has become a major cause of infertility due to a large global trend to delay childbearing.

Through a series of our previous clinical, translational, and laboratory studies, we reported the function of *BRCA1/2* in the maintenance of oocyte reserve in human and mouse ovaries [2–4]. *BRCA1/2* genes carry critical functions in ataxia telangiectasia mutated (ATM) -mediated DNA double-strand break (DSB) repair pathway (ATM-Pathway) and their mutations are associated with predisposition to numerous cancer types [5]. Our original findings of diminished ovarian reserve, indicated by lower serum AMH levels in women with germline *BRCA* mutations have now been further confirmed by a recent individual patient data meta-analysis from five centers worldwide [6]. In a collaborative study, we also observed that *BRCA* mutation carriers experience menopause at an earlier age compared to the general population [7]. The association with earlier menopause and the presence of BRCA mutations has also been independently confirmed by other clinical studies [6–11].

We previously showed that primordial follicle loss and oocyte DNA DSB accumulation are accelerated in the ovaries of humans and mice with *BRCA* mutations [2, 12]. We also showed in single human and mouse oocytes that the gene and protein expression of key ATM-Pathway members decline with age, suggesting that the age-induced DNA DSB accumulation is due to declining DNA DSB repair efficiency. Moreover, we found that women with *BRCA* mutations experience larger ovarian reserve loss and a higher risk of amenorrhea compared to controls in response to genotoxic stressors such as chemotherapy [13, 14]. Cumulatively, these original findings established a central role for *BRCA1* function and the ATM-Pathway in the maintenance of primordial follicle reserve, and generated a novel hypothesis that an age-induced decline in the ATM-Pathway function may contribute and explain the hallmarks of oocyte aging under a single mechanism [2].

Our hypothesis not only explains the age-related accelerated loss of primordial follicles due to declining ATM-mediated DNA DSB repair causing the accumulation of DSBs which trigger apoptotic death, but it also provides a mechanism for age-induced meiotic dysfunction contributing to elevated aneuploidy rates. Oocytes use the same DNA repair mechanisms to mend DSBs that naturally occur during meiotic recombination via Homologous Recombination (HR) [15] between the sister chromatids [16]. While meiotic HR increases genetic diversity, there is evidence that crossovers, which are associated with HR events, also play a stabilizing role in the genome [17, 18]. Recent data suggested the importance of accurate recombination distribution to facilitate the appropriate sequential release of sister chromatid cohesion [19]. Cohesins regulate sister chromatid cohesion, and recent evidence indicates that they migrate to DSB repair sites independently of the normal replication cycle [20]. In addition, convincing evidence has been presented that weakened centromere cohesion is a leading cause of age-related aneuploidy in oocytes [21]. In support of our hypothesis, a cohesin subunit SMC1 is modulated by ATM via phosphorylation and also plays a role in DNA DSB repair [21]. In addition, cohesin subunit SMC1 associates with mitotic microtubules at spindle poles [22]. Finally, it has been shown that *BRCA1* interference results in perturbed spindle and chromosome misalignment in mouse oocytes and that *BRCA1* is required for meiotic spindle assembly and spindle assembly checkpoint (SAC) activation [23].

These findings suggest a key regulatory role for the ATM-Pathway in the meiotic process. Thus, we speculated that the age-related increase in meiotic division errors in oocytes is another symptom of altered ATM-Pathway function, resulting in metaphase plate instability, chromosomal nondisjunction, and age-related aneuploidy [24].

However, direct evidence linking ATM-Pathway dysfunction to diminished oocyte quality is lacking. We performed this study to determine the role of ATM-mediated DNA DSB repair in the maintenance of oocyte quality and to establish a cause-effect relationship between reduced ATM function and impaired oocyte quality. We hypothesized that impairment of oocyte ATM function would compromise oocyte quality and reduce resistance to genotoxic stress. We studied this hypothesis in an oocyte ATM-knockdown (KD) model, coupling it with parthenogenesis, aneuploidy assessment, and a genotoxicity assay. We found that interference with the ATM function in GV oocytes impairs oocyte quality and viability.

## Results

### ATM knockdown decreases *ATM* mRNA and protein expression in mouse oocytes

After the retrieval of mouse ovaries, GV oocytes were microinjected with either 5–10 pl of mouse scrambled siRNA or *ATM* siRNA (injected negative control). A group of oocytes was also cultured without microinjection as an uninjected negative control (UI). Microinjected oocytes were cultured for 24 h to allow enough time for RNA interference and then were assessed for knockdown efficiency.

**Fig. 1:**
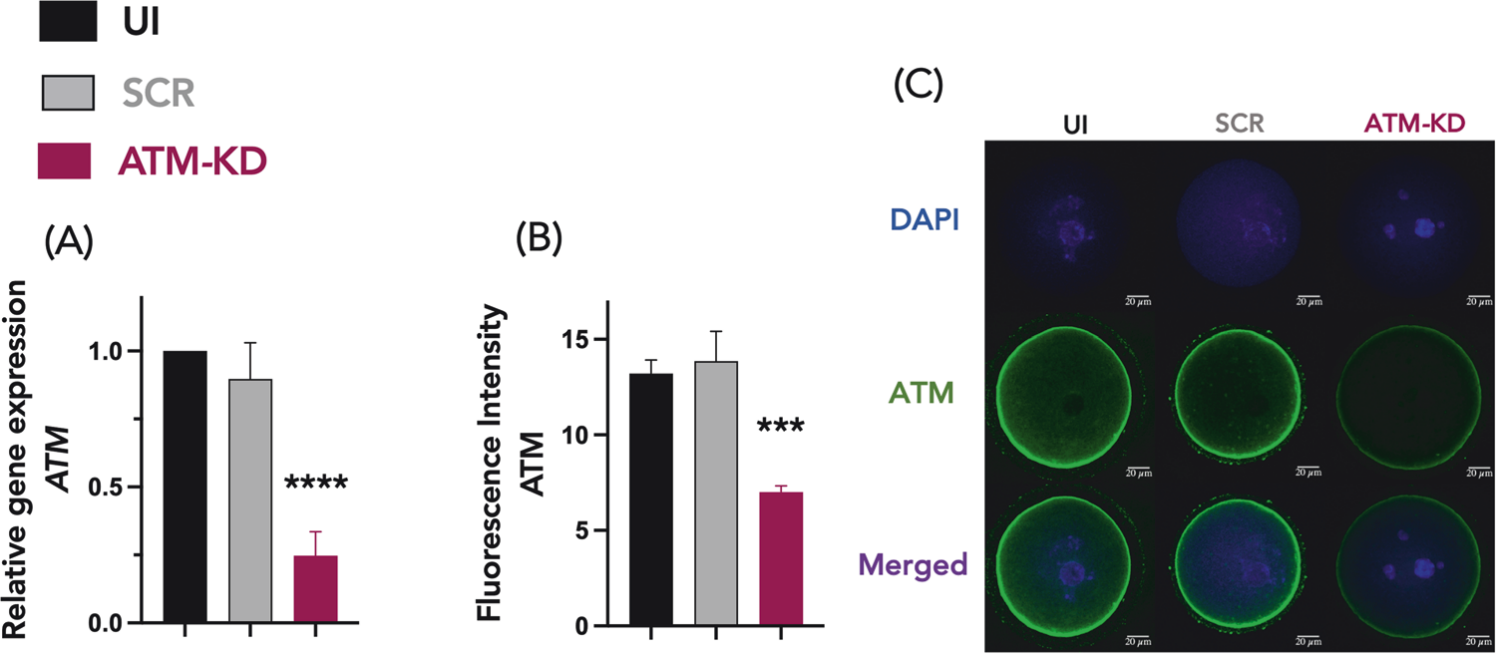
ATM knockdown decreases ATM mRNA and protein expression. Confirmation of the efficacy of RNA interference with *ATM* siRNA in mouse oocytes by microinjection. *ATM* siRNA injection effectively reduced *ATM* gene and protein expression in mouse oocytes. The experiments were replicated a minimum of five times. **A** The bar graph shows a significantly lower relative *ATM* expression in the ATM knockdown (ATM-KD) group, compared to Uninjected (UI) and Scrambled (SCR) controls (oocytes from 6–8 weeks old mice). ****p < 0.0001, Kruskal–Wallis test. *n* = 38 in ATM-KD group, *n* = 27 in SCR, *n* = 38 in UI. Results in mean ± SEM. A.U. (arbitrary units). **B** The bar graph shows a significantly lower ATM fluorescence intensity in the ATM-KD group, compared with the UI and SCR controls. ***p < 0.0005, One-way ANOVA test. *n* = 14 in ATM-KD group, *n* = 18 in SCR, *n* = 35 in UI. Results in mean ± SEM. AU arbitrary units. **C** Representative confocal laser scanning microscope images illustrate lower ATM protein expression in mouse oocytes after ATM knockdown. Cytoplasmic ATM protein expression is shown in green, and the oocytes were counterstained with DAPI in blue.

We first performed qPCR for *ATM* mRNA in each group and the results were analyzed by the ∆∆Ct method. The ATM-KD group showed significantly lower relative *ATM* expression compared to controls (0.25 ± 0.09 vs. 0.90 ± 0.13 in SCR, 1.00 ± 0.00 in UI, *p* < 0.0001 for both, Kruskal–Wallis test) (Fig. 1A). Next, we performed immunofluorescence staining for ATM protein, and the mean fluorescence intensity was measured in each oocyte by a laser scanning confocal microscope. We found that the ATM knockdown resulted in significantly lower ATM fluorescence intensity vs. the controls (7.00 ± 0.34 vs. 13.86 ± 1.53 in SCR, 13.21 ± 0.71 in UI, *p* < 0.0005 for both, one-way ANOVA) (Fig. 1B, C). These results indicate that *ATM* siRNA microinjection effectively downregulates ATM function in our model.

### ATM knockdown impairs meiotic progression in vitro in mouse oocytes

**Fig. 2:**
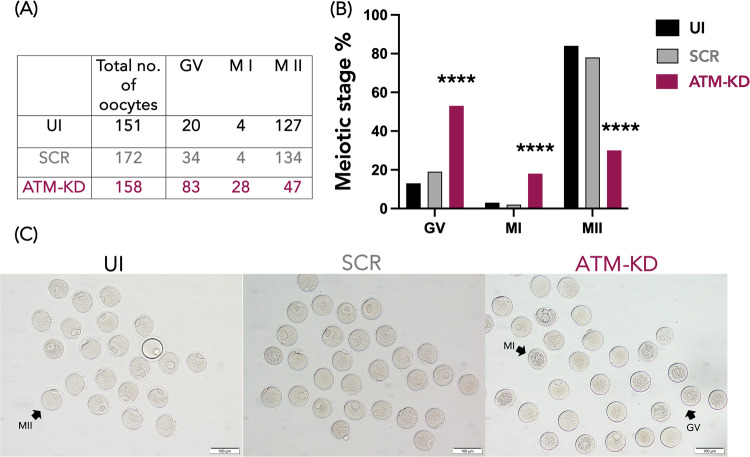
ATM knockdown reduces meiotic progression rate in vitro. The role of ATM function in meiotic progression. ATM knockdown reduced meiotic progression rates in vitro in mouse oocytes. The results were obtained from ten independent experiments. **A** The table shows the total number of oocytes that were subjected to in vitro maturation (IVM) in the UI, SCR, and ATM-KD groups. The meiotic stage was evaluated after 16–20 h of in vitro maturation culture and the oocyte stages were classified as Germinal Vesicle (GV), MI, and MII. **B** The bar graphs represent the distribution (percentage) of meiotic stages in the UI, SCR, and ATM-KD groups after IVM, calculated based on the data in table (**A**). The percentage of immature (GV and MI) oocytes was significantly increased, while the percentage of mature (MII) oocytes was significantly decreased in the ATM-KD group, compared to the UI or SCR controls. ****p < 0.0001, Chi-square test. **C** Representative images of oocytes after IVM in the UI, SCR, and ATM-KD groups, captured under the light microscope (OLYMPUS IX73, 20x magnification). Representative oocytes in different meiotic stages were marked by black arrows. GV germinal vesicle, MI metaphase I, MII: metaphase II.

Both injected and UI oocytes were cultured for 24 hours before being transferred to an IVM medium for 16–20 h, and then they were assessed for meiotic progression. A higher percentage of oocytes were arrested at GV and MI stages after IVM in the ATM-KD group (52.5 vs. 19.8% in SCR and 13.2% in UI for GV stage, 17.7 vs. 2.3% in SCR and 2.6% in UI for MI stage, *p* < 0.0001 for both, Chi-square test), resulting in a lower percentage of oocytes reaching the MII stage compared to controls (29.7 vs. 77.9% in SCR and 84.1% in UI, *p* < 0.0001 for both, Chi-square test) (Fig. 2A–C). As previously suggested in the ovaries of ATM knockout mice lacking primordial and maturing follicles and oocytes [25], this shows that ATM-Pathway function plays a fundamental role in meiotic function.

### ATM knockdown impairs mouse embryo cleavage, blastocyst formation and morphology

**Fig. 3:**
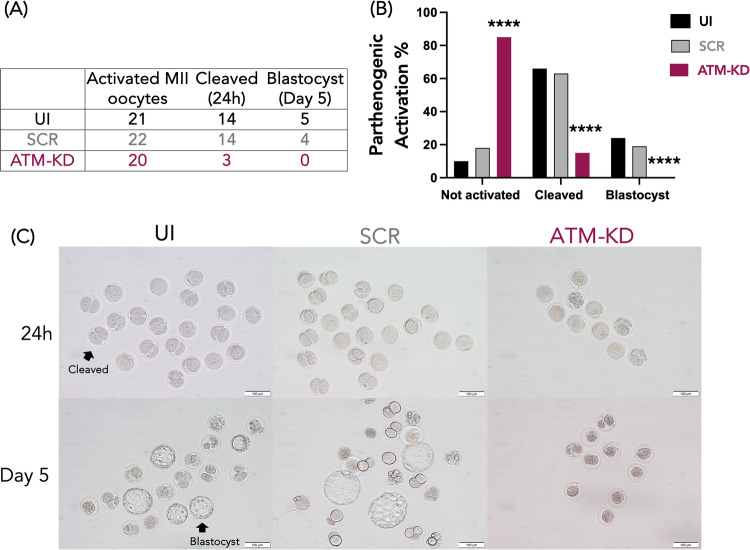
ATM knockdown reduces embryo cleavage and blastocyst formation. The role of ATM function in embryo development and quality. ATM knockdown resulted in reduced cleavage and blastocyst formation, and poor embryo morphology. The experiments were replicated at least four times. **A** The table shows the total number of mature (MII) oocytes that were subjected to parthenogenic activation for 4 h in the UI, SCR, and ATM-KD groups, and raw results after 24 h (Cleaved) and 5-day (Blastocyst) culture. **B** The bar graphs show the parthenogenic activation rates and the distribution of embryonic stages in the UI, SCR, and ATM-KD groups calculated based on the data in table (**A**). The parthenogenic activation rate was calculated as the ratio of cleaved embryos to the total number of activated MII oocytes. A significantly higher percentage of oocytes failed to parthenogenically activate after ATM knockdown compared to UI and SCR controls. The percentage of embryos reaching the cleavage and blastocyst stage were significantly lower in the ATM-KD compared to the UI and SCR controls. ****p < 0.0001, Chi-square test. **C** Light microscopic images representing the parthenogenic activation process at 24 h and 5 days in the UI, SCR, and ATM-KD groups (20x magnification). No MII oocyte developed into blastocyst stage after ATM knockdown and all displayed poor embryo morphology.

Following IVM from the GV stage, MII oocytes were cultured in parthenogenic activation media for 4 h and then placed in the culture medium to observe embryo development. This resulted in very few oocytes cleaving and none reaching the blastocyst stage after the ATM knockdown (Fig. 3A). The ATM knockdown also significantly reduced the percentage of cleaved oocytes and blastocyst formation, compared to controls (15.0 vs. 63.6% in SCR and 66.7% in UI for cleaved rate, 0.0 vs. 18.1% in SCR and 23.8% in UI for blastocyst rate, *p* < 0.0001, Chi-square test) (Fig. 3A, B). The embryos that were able to cleave after ATM knockdown were of poor morphology (Fig. 3C). These findings support that ATM function is critical in the regulation of embryo development and quality.

### ATM knockdown is associated with increased aneuploidy

**Fig. 4:**
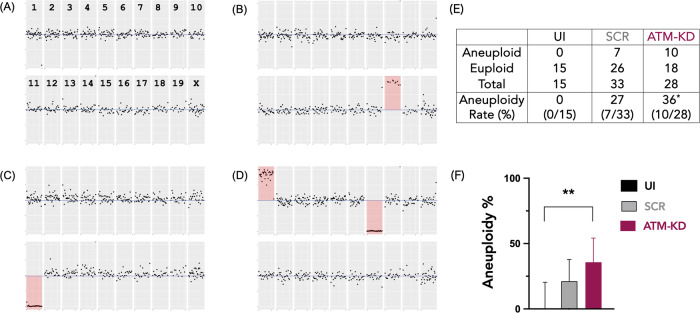
ATM knockdown is associated with a higher rate of aneuploidy. The impact of ATM knockdown on aneuploidy incidence. One-pronucleus (1-PN) parthenotes in the UI, SCR, and ATM-KD groups were subjected to low-pass genome sequencing to determine the impact of ATM knockdown on aneuploidy rates. The pictures are examples of cytogenic results interpreted from low-pass genome sequencing. The relative amount of DNA from each chromosome (divided into 5 Mb segments) was assessed with respect to the amounts seen in chromosomally normal reference samples. Chromosome with altered copy number are highlighted in red. As a result, ATM knockdown increased the baseline aneuploidy rate. The results were obtained from ten independent experiments. The picture shows (**A**) the result from an embryo with an equal number of copies of each chromosome; **B** Embryo with an extra copy of chromosome 18; **C** Embryo lacking chromosome 11; **D** Embryo with an extra copy of chromosome 1 and loss of chromosome 7. **E** The table shows the raw results of the aneuploidy analysis from 1-PN parthenotes in each group. The aneuploidy rate is calculated as the ratio of aneuploid to the total number of 1-PN parthenotes analyzed. **F** The bar graphs show aneuploidy % in the UI, SCR, and ATM-KD groups, based on the data in (**A**). The ATM-KD was associated with a significantly higher aneuploidy rate compared to the UI controls. **p < 0.01, Fischer’s exact test. The ATM-KD also resulted in an increase in aneuploidy rates compared to the SCR, but this difference did not reach statistical significance. However, the odds ratio for aneuploidy was 2.0 (95% CI 0.58–7.6, Fischer’s test) after ATM knockdown compared to the SCR controls.

A group of parthenogenic embryos (*n* = 76) representing the ATM-knockdown and control groups were collected at the 1-cell (pronucleus) stage and were subjected to low-pass genome sequencing to determine aneuploidy rates. We found that, ATM knockdown was associated with a significantly increased frequency of aneuploidy compared to UI controls (36% vs. 0% in UI *p* < 0.01, Fischer’s test) (Fig. 4A). The odds ratio of developing aneuploidy compared to controls injected with a scrambled siRNA (SCR) was 2-fold higher after the ATM knockdown (95% CI of 0.58–7.6, Fischer’s test) (Fig. 4B), though this difference did not reach statistical significance given the sample size restrictions (please see discussion). These findings indicate that while the microinjection process might have contributed to it, ATM knockdown independently results in increased aneuploidy in embryos.

### ATM knockdown increases DNA DSBs in response to genotoxic stress

To assess the role of ATM in protecting oocytes against genotoxic stress, we exposed both injected and UI oocytes to 4-HC for 4 h which were then placed in plain culture media. The oocytes were then assessed for DNA DSBs by γH2AX immune-fluorescence staining at 0, 6, and 12 h after the completion of 4-HC exposure.

**Fig. 5:**
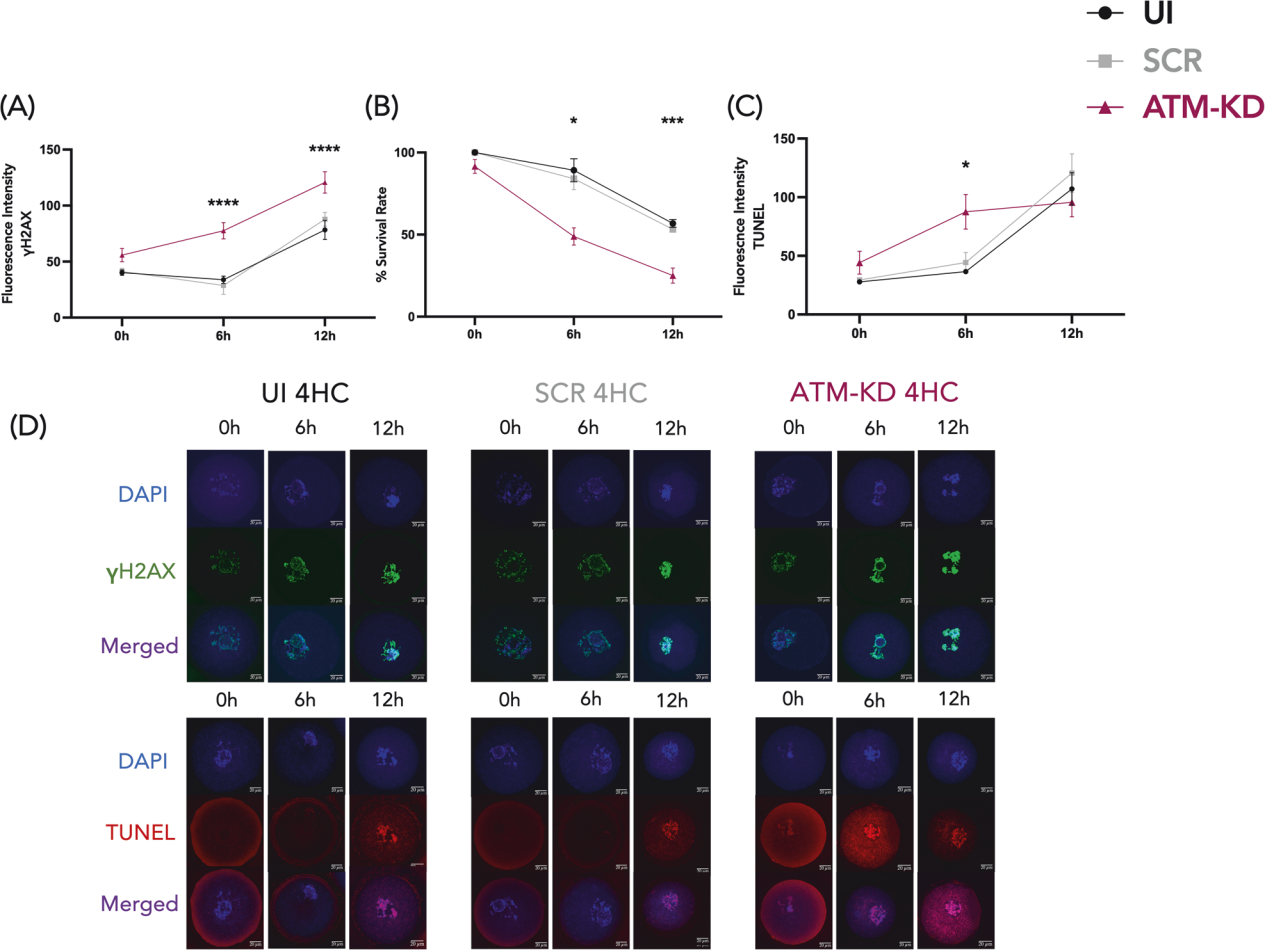
ATM knockdown increases DNA DSBs in response to genotoxic stress. The role of ATM function in oocyte genomic integrity and survival after genotoxic stress. To assess the role of ATM in protecting oocytes against genotoxic stress, we exposed both injected and UI oocytes to 4-HC for 4 h. The ATM-KD resulted in a greater accumulation of DNA DSBs and earlier apoptotic death compared to controls in response to genotoxic stress. The experiments were replicated at least 3 times at each timepoint. **A** The line graph shows the quantification of γH2AX fluorescence intensity in mouse oocytes at 0, 6, and 12 h culture timepoints after 4-HC treatment. The 4-HC treatment significantly increased the γH2AX fluorescence intensity in the ATM-KD group, compared to the UI and SCR controls at 6 and 12 h, ****p < 0.0001, one-way ANOVA test. ATM-KD, *n* = 15, *n* = 16, *n* = 15, at 0, 6, and 12 h timepoints respectively, SCR, *n* = 14, *n* = 11, *n* = 21, at 0, 6, and 12 h timepoints respectively, UI, *n* = 9, *n* = 18, *n* = 16, at 0, 6, and 12 h timepoints respectively. Results are in mean ± SEM. **B** The line graph shows the % survival rates after the 4-HC exposure in mouse oocytes at 0, 6, and 12 h of culture timepoints. In the ATM-KD group at 6 h, the 4-HC treatment resulted in a significantly lower survival rate compared to the UI. *p < 0.03, Kruskal–Wallis test. *n* = 42 in ATM-KD, *n* = 38 in SCR, *n* = 54 in UI. At 12 h, ATM-KD resulted in a lower survival rate compared to both the UI and SCR-4-HC treated controls, ***p < 0.0005, Kruskal–Wallis test. *n* = 74 in ATM-KD, *n* = 28 in SCR, *n* = 59 in UI. Data shown as mean ± SEM. **C** The line graph shows the quantification of TUNEL fluorescence intensity at 0, 6, and 12 h of culture timepoints after the 4-HC treatment. ATM knockdown resulted in an earlier manifestation of apoptosis after 4-HC exposure at 6 h compared to UI and SCR controls. *p < 0.03 for UI, **p < 0.01 for SCR, Kruskal–Wallis test. *n* = 16 in ATM-KD, *n* = 17 in SCR, *n* = 17 in UI. Data were in mean ± SEM. **D** The representative confocal laser scanning microscope images of γH2AX and TUNEL staining in mouse oocytes after 4-HC treatment. Nucleic γH2AX staining is shown in green and nucleic TUNEL staining is shown in red, while the oocytes were counterstained with DAPI in blue. The images show an earlier increase in DNA DSBs followed by earlier apoptotic death in the ATM-KD after 4-HC exposure.

The 4-HC exposure resulted in a significant increase in DNA DSBs in the ATM*-*KD group compared to the 4-HC exposed UI or SCR controls at 6 and 12 h timepoints, (77.6 ± 7.3 vs. 28.7 ± 7.9 in SCR-4-HC at 6 h, 33.8 ± 3.3 in UI-4-HC at 6 h, *p* < 0.0001 for both, one-way ANOVA. 120.7 ± 9.5 vs. 87.8 ± 6.1 in SCR-4-HC at 12 h and 78.3 ± 8.5 in UI-4-HC at 12 h, *p* < 0.0001, one-way ANOVA, Fig. 5A). Overall, ATM knockdown resulted in the highest magnitude of DNA DSB accumulation foci among all groups, at all of the timepoints studied (Fig. 5A).

### ATM knockdown induces earlier apoptotic death in mouse oocytes after genotoxic exposure

To determine if the observed increase in DSBs after ATM knockdown was associated with reduced oocyte viability, we assessed oocyte survival rates at 0, 6, and 12 h timepoints. We found that in the ATM-KD group, 4-HC exposure significantly reduced oocyte survival compared to the UI at 6 h (48.9 vs. 89.2% in UI, *p* < 0.03, Kruskal–Wallis test. Fig. 5B). Although not reaching statistical significance, ATM knockdown also resulted in lower survival rates than SCR at 6 h (48.9 vs. 84.0% in SCR, *p* = 0.06, Fig. 5B). At 12 h, compared to both UI and SCR, 4-HC exposure significantly reduced oocyte survival in the ATM-KD group (25.0 vs. 53.2% in SCR, *p* < 0.003, 56.8% in UI, *p* < 0.0005, Kruskal–Wallis test, Fig. 5B).

Next, we performed a TUNEL assay to confirm that the reduced oocyte survival rates were associated with increased apoptosis. The 4-HC exposure resulted in earlier accumulation of TUNEL fluorescence intensity in the ATM-KD compared to the UI and SCR at 6 h (87.5 ± 14.8 vs. 44.3 ± 8.5 in SCR, 36.5 ± 2.0 in UI, *p* < 0.03, Kruskal–Wallis test., Fig. 5C). At 12 h, TUNEL fluorescent intensity in the UI and SCR caught up with the ATM-KD after 4-HC exposure. These findings indicate that ATM plays an active role in oocyte defense against acute genotoxic stress and its functional decline may result in liability to genotoxic agents triggering apoptotic death.

## Discussion

In this gene manipulation study with mouse oocytes, we found that downregulation of ATM function results in impaired meiotic progression and poor embryo development in vitro, with a tendency for increased aneuploidy. Moreover, we found that ATM knockdown causes increased susceptibility to genotoxic agents with reduced oocyte survival and increased DNA DSB formation and apoptosis. These findings mimic age-related changes in the human ovary, and indicate an important role for ATM in the maintenance of oocyte quality. Taken together with our prior research showing an age-induced decline in ATM function in mouse and human oocytes [2, 26, 27], this is the first direct evidence supporting the notion that this decline may be a prime factor contributing to ovarian aging.

In affirmation of our clinical and translational laboratory studies supporting a central role of declining ATM-Pathway function in ovarian aging, a large GWAS study in ~70,000 women found genes related to DNA DSB repair, especially belonging to the ATM-Pathway, to be critical in determining age at natural menopause [28]. A later GWAS study involving nearly 200,000 women from the same group found associated variants in a broad range of DNA damage response (DDR) genes at normal natural menopause, *BRCA1*, a key member of the ATM-Pathway having the strongest relationship [29].

We have, therefore, previously hypothesized that declining ATM-Pathway function can explain both the age-induced follicle loss and declining oocyte quality under a single mechanism. We surmise that two simultaneous processes occur during aging in oocytes: decreased ATM-Pathway function results in loss of ovarian reserve by the accumulation of lethal DSBs, while the same alteration increases aneuploidy via disrupted meiotic function [27]. Our study provides novel evidence linking declining ATM-Pathway function to meiotic dysfunction and aneuploidy.

Errors in either the first or second meiotic division lead to embryonic aneuploidies [30]. Notably, 75% of serious chromosome errors in human oocytes occur in meiosis-I [31]. The loss of cohesin and spindle defects have been put forward as the two most direct contributing factors to meiotic chromosome/chromatid segregation errors [32, 33]. The expression and function of cohesin is substantially reduced in both human and mouse oocytes with age, destabilizing chiasmata that help hold sister chromatids together [34, 35].

Cohesin is composed of several subunits, including SMC1, the function of which is modulated by ATM-Pathway-mediated phosphorylation [21, 23, 36, 37]. Recent evidence has shown that cohesin also contributes to ATM-mediated DNA damage signaling and repair [38]. Furthermore, other recent studies indicated that Smc1β is essential for activation of SAC during mouse oocyte meiosis [39, 40] and associates with mitotic microtubules at spindle poles [22]. It has also been shown that *BRCA1* is required for chromosomal alignment and spindle formation; when *BRCA1* is silenced, mouse oocytes show meiotic perturbation [23]. These findings support that ATM may regulate the fidelity of the meiotic process through multiple members of its pathway.

In our study, we also found that ATM knockdown results in increased sensitivity to genotoxic DNA damage and apoptotic death in mouse oocytes. Though the ATM-Pathway appears to have a critical role in repairing damage induced by genotoxic stress [2, 26, 27], such repair has its limitations and can be overwhelmed with intense damage [27, 41]. To enable successful repair, cells can tolerate a large number of DNA DSBs before triggering cell death mechanisms [41]. There is likely a threshold of DSBs that has to be crossed before oocytes “give up” on repair [27]; thus, a balance between the severity of the ovarian damage caused by chemotherapy and the ability of the oocytes to repair has been suggested [27].

Shortly after the formation of DNA DSBs, the MRE11, Rad50, and NBSI (MRN) complex and γH2AX are attracted to the DNA DSB sites to facilitate repair [27]. Because γH2AX binds to DNA DSB sites in a 1:1 manner [42], it can be used to quantify the level of DNA damage [43]. If the damage is beyond repair, ATM activates TAp63 either directly or indirectly via CHK1/CHK2 to induce apoptosis [44–51]. Our and others’ xenografting and/or in vitro culture studies clearly showed by both TUNEL and AC3 expression that chemotherapy exposure (including cyclophosphamide and doxorubicin) causes massive primordial follicle oocyte apoptosis [26, 44, 52–58]. The co-expression of AC3 and γH2AX in chemotherapy-exposed follicles suggested that unrepaired DSBs were resulting in apoptotic death [26]. Recent evidence is highly supportive of the notion that gonadotoxic chemotherapy results in acute and massive depletion of primordial follicle reserve by direct damage to the DNA and resultant apoptosis [44]. In our study, ATM knockdown resulted in reduced survival and increased apoptotic death in mouse oocytes, indicating that ATM has an important function in defending oocytes against genotoxic stress. The ATM-knockdown oocytes died earlier than those in the control groups after the chemotherapy exposure, as determined by both morphological survival criteria and apoptosis intensity using TUNEL. In parallel, while DNA DSBs rapidly accumulated in the ATM-knock-down oocytes after the exposure to 4-HC, the DNA DSB accumulation did not significantly increase until 12 h after the genotoxic exposure in the ATM-intact controls. These findings indicate that ATM plays an active role in oocyte defense against acute genotoxic stress and its functional decline may result in liability to genotoxic agent-triggered DNA damage and apoptotic death. Our findings also indicate that, even in oocytes with intact ATM function, DNA damage will accumulate with time as the threshold for repair is exceeded.

In the ATM-Pathway, an alternative oocyte fate to apoptotic death or DNA repair and survival after encountering genotoxic stress is cell senescence. While we did not study cell senescence in the current study, we previously showed that pharmacological inhibition of ATM rescues oocytes from doxorubicin-induced death but does not prevent them from arresting in the M-I phase. This is likely due to oocyte senescence preventing the propagation of severe mutagenesis [26]. In fact, irreparable DNA damage from chemotherapy has been put forward as one of the several key mechanisms of senescence in reproductive cells [59].

While our study puts forward a novel hypothesis that the weakening of ATM-Pathway function is a critical contributor to oocyte aging, the data on the increased aneuploidy rate is limited by the sample size. Though we found a statistically significant increase in aneuploidy rate after ATM knockdown compared to UI controls and the odds ratio for aneuploidy was two-fold higher compared to the SCR-injected control group. However, this difference did not reach statistical significance for the latter comparison. This suggests that the impact of ATM knockdown on spindle function might have been compounded by the microinjection process itself. Our power analysis indicates that a large number of parthenotes will be needed to detect smaller differences in the risk of aneuploidy between the ATM-knockdown and SCR-injected control groups. Given the low incidence of aneuploidy in mouse oocytes, however, the ability to induce a significant aneuploidy risk in parthenotes derived from ATM-knockdown oocytes suggests a critical role for ATM in maintaining mouse oocyte meiotic integrity. This conclusion is particularly supported by our findings of meiotic arrest in vitro, poor embryo development and lack of development to blastocyst stage after ATM knockdown in comparison to both SCR-injected and UI control groups.

Given the shared role of the ATM-Pathway regulated DNA repair efficiency in oocyte aging and chemotherapy-induced germ-cell loss, we speculate that future treatments enhancing ATM-Pathway function or targeting components of that pathway to regulate repair, apoptotic death or senescence may prevent, retard or reverse ovarian aging, and may be a fertility preservation strategy against gonadotoxic chemotherapy. We have shown parallel findings between our mouse and human ovarian tissue models in previous studies [2], and the ATM knockdown replicates the clinical findings of oocyte aging in women. Therefore, the findings from the current mouse study are promising for human translation and bringing future targeted drug discoveries to curb reproductive aging.

## Materials and methods

### Mouse oocyte collection and RNA interference

Friends Leukemia Virus B (FVB) female mice (Taconic Bioscience, New York, United States) of 6–8 weeks age were stimulated by intraperitoneal injection with 10 IU of pregnant mare serum gonadotropin (PMSG) (Reference (REF): HOR-272-a, ProSpec, Israel), 48 h prior to geminal vesicle (GV) stage oocyte collection. After removing cumulus cells by pipetting, the retrieved oocytes were microinjected with either 50 µM of scrambled (SCR) (REF: sc-36869, Santa Cruz Biotechnology, Inc., Texas, USA) or *ATM* siRNA (REF: sc-29762, Santa Cruz Biotechnology, Inc.) in modified HTF medium (REF: 90126, FUJIFILM, Japan) supplemented with 0.1 mM 3-isobutyl-1-methylxanthine (IBMX) (REF: I5879, Sigma-Aldrich, Missouri, United States). A group of oocytes was also cultured without microinjection (uninjected controls, UI). Microinjected and UI oocytes were cultured in Minimum Essential Medium α (αMEM) with GlutaMAX (REF: 32571-036, Gibco, Massachusetts, USA) supplemented with 4 mg/ml bovine serum albumin (BSA) (REF:12657, EMD Millipore Corporation, Massachusetts) and 0.1 mM IMBX for 24 hours. The efficiency of knockdown was confirmed by qPCR for the *ATM* mRNA and by Leica SP8 confocal microscope (Leica Microsystems, Germany) for the ATM protein.

Our animal experiments were conducted in accordance with the guidelines of the Institutional Animal Care and Use Committee of Yale University.

### Single-cell real-time qPCR

RNA from single GV oocytes was extracted by dissolving to lysis buffer with 0.2% (vol/vol) Triton X-100 (REF: 161–0407, Bio-Rad Laboratories, Inc., California, United States). Reverse transcription was conducted by using the Super Script IV reverse transcriptase (REF: 18090050, Invitrogen, Massachusetts, USA) according to the kit instructions, followed by 22 cycles of PCR pre-amplification using KAPA HiFi HotStart ReadyMix (REF: 07958897001 Roche Diagnostic Corporation, Indianapolis, United States). PCR amplification cycles were subsequently carried out by using PowerUp SYBR Green Master Mix (REF: A25742, Applied Biosystems, Massachusetts, United States) and were detected on the ABI QuantStudio-6 Flex Real-Time PCR machine. The mouse β-actin primer was used as a control to *ATM* primer. The relative gene expression was calculated by using the ΔΔCt method. The primer sequences were as follows:

5’-GGCTGTATTCCCCTCCATCG-3’ (The forward primer of mouse β-actin),

5’-CCAGTTGGTAACAATGCCATGT-3’ (The reverse primer of mouse β-actin),

5’-TGGGTGGACAGGTGAACTTGCT-3’(The forward primer of mouse *ATM*),

5’-ACCCAAGCTTTCCATCCTGGGA-3’(The reverse primer of mouse *ATM*),.

### Immunofluorescence staining

After the culture, the oocytes were collected in 0.1% polyvinylpyrrolidone (PVP) phosphate-buffered saline (PBS) for washing, and they were immediately fixed with 4% paraformaldehyde in PBS for 30 minutes at room temperature. After permeabilization in 0.1% Triton X-100 in PBS for 10 min at room temperature, the oocytes were transferred to a blocking buffer (3% BSA and 5% Normal Donkey Serum (NDS) in PBS) for overnight incubation at 4 °C. The primary antibodies and the concentration that were used in this study were followings: purified anti-H2A.X Phospho (Ser139) (REF: 613401, Biolegend, California, United States) in 1:500 and Terminal deoxynucleotidyl transferase dUTP Nick End. Labeling (TUNEL) (REF: 12156792910, Roche, Switzerland) with 2U of the enzyme blend in 50 µl. The secondary antibody was incubated in a blocking buffer without NDS at 1:500 for an hour. Alexa Fluor 488 goat anti-mouse (REF: A11029, Invitrogen) was used as a secondary antibody for anti-H2A.X Phospho. After washing with 0.1% PVP PBS, the oocytes were mounted onto a slide with ProLong Gold antifade reagent with DAPI (REF: P36935, Invitrogen). The slides were left to dry for 24 h and were then scanned by Leica TCS SP8 confocal laser scanning microscope (Leica Microsystems).

### In vitro maturation and parthenogenic activation

Both injected and UI oocytes were cultured for 20 h to allow enough time for RNA interference. Those matured in vitro were parthenogenically activated, while a group of embryos was allowed to develop to test the impact of ATM knockdown on embryo development.

### 4-HC treatment and DNA damage assessment

A group of injected and UI oocytes were incubated with 20 µg/ml of 4-hydroxy-cyclophsphamide (4-HC) for 4 h. They were then assessed for DNA DSBs by immune-fluorescence staining using anti-γH2AX phosphor-antibody at 0-, 6-, and 12-h timepoint after the exposure. Both of the control and 4-HC treated groups of oocytes, including injected and UI were collected in 0.1% PVP PBS in each designated well for washing. They were immediately fixed with 4% paraformaldehyde in PBS for 30 min at room temperature. After permeabilization in 0.1% Triton X-100 in PBS for 10 min at room temperature, the oocytes were transferred to a blocking buffer (3% BSA and 5% Normal Donkey Serum (NDS) in PBS solution) for overnight incubation at 4 °C. The purified anti-H2A.X Phospho (Ser139) (REF: 613401, Biolegend) was used as the primary antibody in 1:500. After washing in 0.1% PVP PBS, the secondary antibody, Alexa Fluor 488 goat anti-mouse (REF: A11029, Invitrogen) were incubated in a blocking buffer without NDS in 1: 500 for an hour. After washing the oocytes with 0.1% PVP PBS, the oocytes were mounted onto a slide with ProLong Gold antifade reagent with DAPI (REF: P36935, Invitrogen). The slides were allowed to dry for 24 h. The slides were scanned by a Leica TCS SP8 confocal laser scanning microscope (Leica Microsystems).

### Detection of apoptosis

After the treatment with 20 µg/ml of 4-HC for 4 h, the oocytes were transferred to αMEM with GlutaMAX, supplemented with 4 mg/ml BSA and 0.1 mM IBMX. Apoptosis was assessed in the oocytes both morphologically and by immunofluorescent staining for TUNEL. The oocytes were assessed by light microscope for survival, after chemotherapy at 0-, 6-, and 12-h timepoints. The oocytes were assessed morphologically for viability. Those oocytes that had a cytoplasmic condensation with retraction of the oolemma from the zona pellucida, budding and cellular fragmentation, with apoptotic bodies (membrane-enclosed vesicles of unequal size) were considered non-viable [60, 61]. Those oocytes that did not have these morphological features were considered viable. The oocyte survival rate was calculated by the number of surviving oocytes divided by the total number of oocytes that were 4-HC treated.

In preliminary experiments, we validated our morphological assessment with 7-aminoactinomycin D (7-AAD) (REF: A1310, Invitrogen) with dead/live staining and showed 100% concordance (See Supplementary Fig. 1). The oocytes were stained with TUNEL to determine apoptotic death at 0-, 6-, and 12-h timepoints.

### Cytogenetic analysis using low-pass genome sequencing

Parthenogenetic embryos were collected at the one-cell stage, after successful activation (indicated by pronucleus formation) but prior to the first mitotic division. The zona pellucida and polar bodies were removed following a brief incubation in acidified Tyrode’s solution, after which each embryo was washed in a droplet of phosphate-buffered saline and placed in a microcentrifuge tube. Embryo cells were lysed, and the DNA was subjected to whole genome amplification (SurePlex; Illumina, California, USA). A low-pass next-generation sequencing approach was used to detect aneuploidy [62], using a protocol previously validated in murine embryos [63]. Briefly, sequencing libraries were created from the amplified DNA using the SQK-LSK-108 kit (Oxford Nanopore Technologies, United Kingdom) and sequencing was undertaken using the MinION device (Oxford Nanopore Technologies). Samples were demultiplexed using the Epi2Me software (Oxford Nanopore Technologies) and adapters and barcodes were trimmed using the Porechop tools. Sequences were aligned to the Mus musculus genome (GRCm38_68) in Minimap2, using the default parameters. Chromosomes were divided into 5 Mb intervals (“bins”) and the proportion of reads within each bin was calculated with reference to the total number of aligned reads. These results were compared to a reference data set composed of data from multiple karyotypically normal female samples.

### Statistical analysis

An average of 15 oocytes were used in each experiment, which was at least triplicate. Data were presented as mean ± SEM. One-way ANOVA and Tukey’s multiple comparison test was used when data followed a normal distribution and homogeneity of variances among groups were assumed. When the data did not follow the normal distribution, a non-parametric test was applied. An asterisk denotes a significant difference in the figures.

### Supplementary information

Supplementary Information

## Data Availability

All data were available in the main text or the supplementary materials.
