# Supplementary material for: The role of declining ataxia-telangiectasia-mutated (ATM) function in oocyte aging
Source: Cell Death Discov. 2024 Jun 25;10:302. doi: 10.1038/s41420-024-02041-z (PMC11196715; doi:10.1038/s41420-024-02041-z)
Supplement: Supplementary file 1 — Supplementary Information [file 41420_2024_2041_MOESM1_ESM.docx]

**Supplementary Information**

Contents

1. Figure S1. Comparison of live/dead staining with morphological assessment of oocyte viability (page1)

2. Figure S2. ATM knockdown causes earlier accumulation of DNA DSBs with greater magnitude after 4-HC exposure (page 2)

3. Figure S3. ATM knockdown causes earlier accumulation of TUNEL after 4-HC exposure (page3)

**
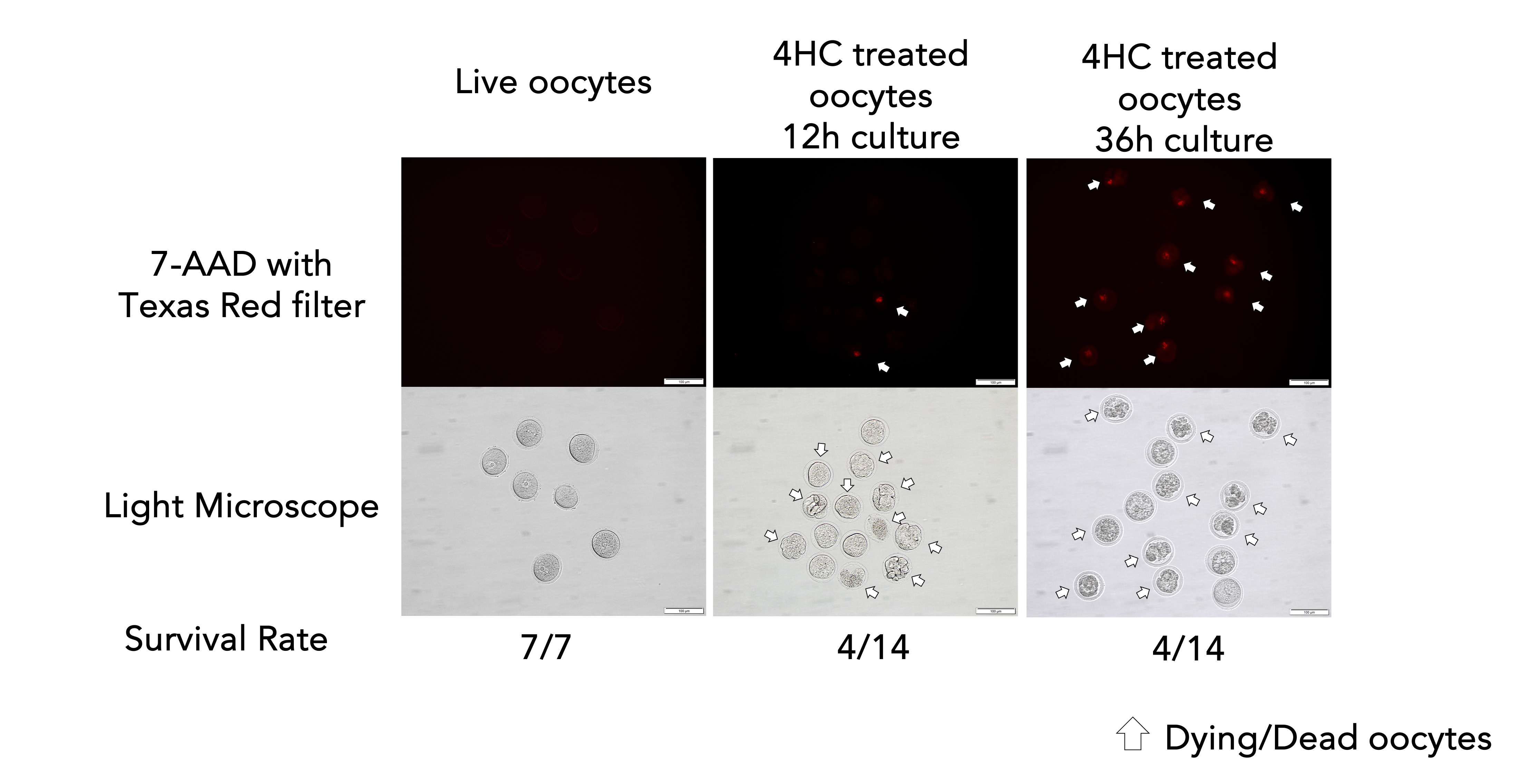
**

Figure S1. Comparison of live/dead staining with morphological assessment of oocyte viability

The representative fluorescent microscopic images of live, dead, and fragmented (dying) oocytes assessed both by 7-AAD and light microscopy. We confirmed that morphological survival assessment correlates with 7-AAD staining. Although oocyte fragmentation is a hallmark of apoptosis and considered as a sign of non-viability by morphological assessment, fragmented oocytes did not pick up 7-AAD until 36h. Hence, 7-AAD assessed survival rate at 36h matched the morphologically assessed survival rate at 12h. The arrows point to matching 7-ADD stained and morphologically assessed “dead” oocytes.

**
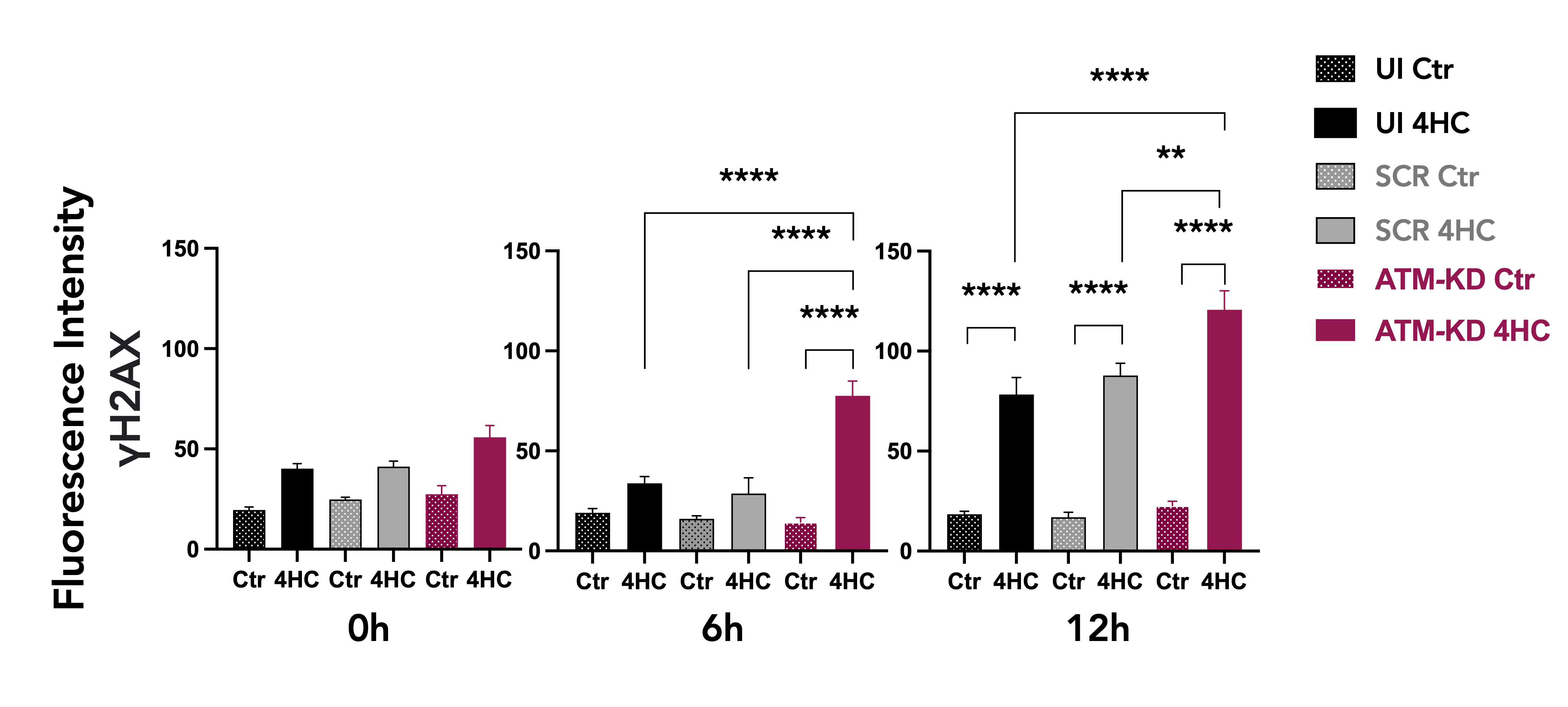
**

Figure S2. ATM knockdown causes earlier accumulation of DNA DSBs with greater magnitude after 4-HC exposure

The bar graphs show the mean intensity of γH2AX in the UI, SCR and ATM-KD groups of with and without 4-HC exposure. We found that ATM knockdown was associated with significantly increased levels of DNA DSBs after 4HC exposure compared to the no 4-HC exposure control as early as at 6h time point (77.6 ± 7.3 vs. 14.5 ± 2.3 in ATM-KD Ctr, **** *p*<0.0001, one way ANOVA) but the same exposure did not result in significant increase in DSB in UI and SCR groups until 12h. Yet, at 12h, the magnitude of DNA DSBs was greater after the 4-HC exposure in the ATM-KD group compared to the 4-HC treated UI and SCR. ** *p* <0.01, *****p*<0.0001, one-way ANOVA. Therefore, ATM knockdown was associated with earlier and higher magnitude of accumulation of DNA DSBs. There was no difference in γH2AX fluorescence intensity without the 4-HC exposure in any of the treatment groups.

ATM-KD Ctr group, n=10, n=14, n=22 at 0, 6, and 12h, respectively. SCR Ctr group, n=8, n=8, n=8, at 0, 6, and 12h, respectively. UI Ctr group, n=19, n=9, n=16 at 0, 6 and 12h, respectively.


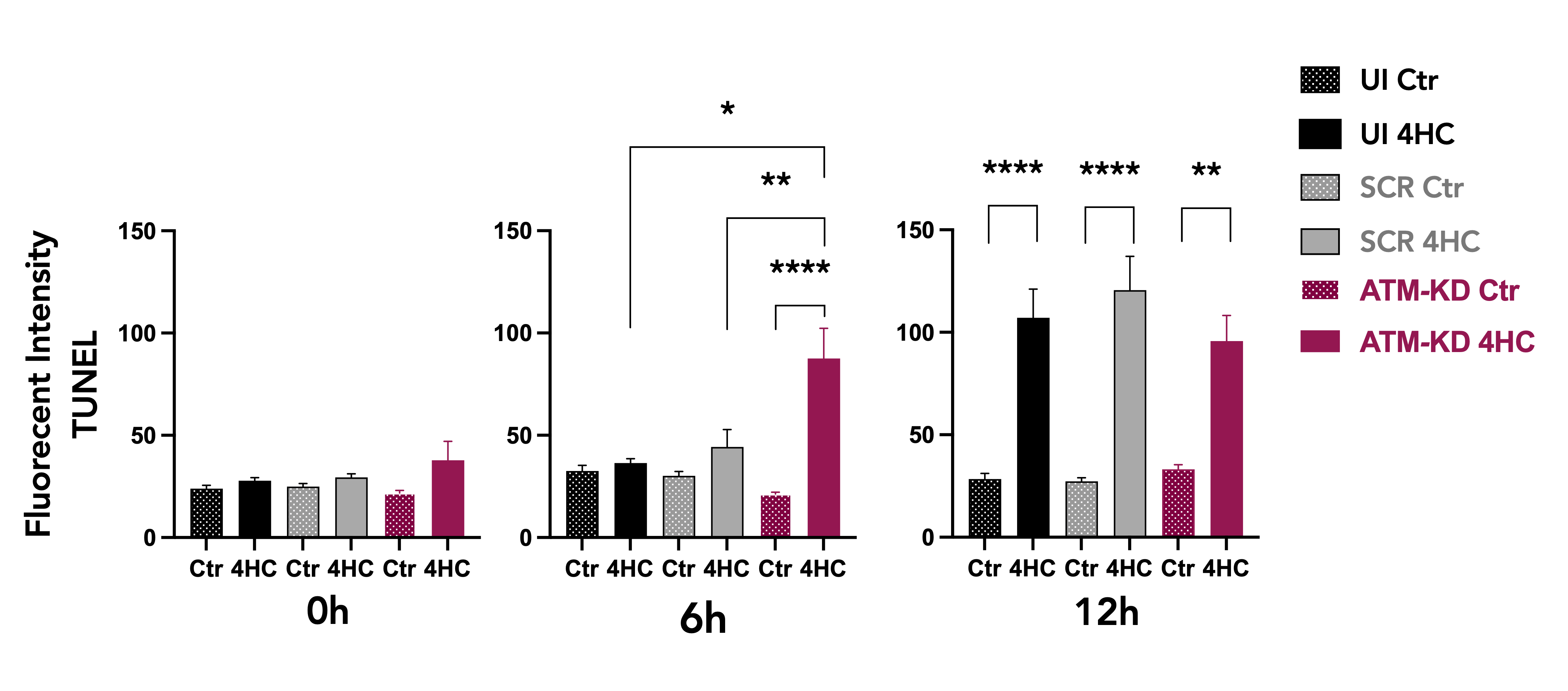


Figure S3. ATM knockdown causes earlier accumulation of TUNEL after 4-HC exposure

The bar graphs show the mean intensity of TUNEL in the UI, SCR and ATM-KD groups with and without the 4-HC exposure. The 4-HC exposure resulted in earlier accumulation of TUNEL at 6h in the ATM-KD compared to the UI and SCR controls. ***p*<0.01 for SCR, **p<*0.03 for UI, Kruskal Wallis test. At 12h, the 4-HC exposure resulted in a similar TUNEL fluorescent intensity among the ATM-KD, UI and SCR 4-HC exposed groups, as the latter caught up with the ATM-KD, 107.1 ± 14.0 in UI Ctr vs. 28.5 ± 2.7 in UI 4-HC, 120.6 ± 16.43 in SCR Ctr vs. 27.3 ±1.7 in SCR 4-HC, *****p*<0.0001 for both, Kruskal Wallis test.

Without the 4-HC exposure, the TUNEL fluorescence intensity did not change over time points in the ATM-KD or control groups. n=14, n=14, n=18 at 0, 6 and 12h, respectively in ATM-KD, n=18, n=15, n=12 at 0, 6 and, 12h, respectively in SCR, n=19, n=14, n=17 at 0, 6 and, 12h, respectively in UI.
